# Bioinertization of NanoLC/MS/MS Systems by Depleting Metal Ions From the Mobile Phases for Phosphoproteomics

**DOI:** 10.1016/j.mcpro.2023.100535

**Published:** 2023-03-22

**Authors:** Yumi Komori, Tomoya Niinae, Koshi Imami, Jun Yanagibayashi, Kenichi Yasunaga, Shinya Imamura, Masami Tomita, Yasushi Ishihama

**Affiliations:** 1 Graduate School of Pharmaceutical Sciences, Kyoto University, Kyoto, Japan; 2 Proteome Homeostasis Research Unit, RIKEN Center for Integrative Medical Sciences, Yokohama, Kanagawa, Japan; 3 Analytical & Measuring Instruments Division, Shimadzu Corporation, Kyoto, Japan; 4 Laboratory of Clinical and Analytical Chemistry, National Institute of Biomedical Innovation, Health and Nutrition, Ibaraki, Osaka, Japan

**Keywords:** bioinert, phosphopeptide, metal-chelating, adsorption, nanoLC/MS/MS

## Abstract

We have successfully developed a bioinertized nanoflow LC/MS/MS (nanoLC/MS/MS) system for the highly sensitive analysis of phosphopeptides by depleting metal ions from the mobile phase. We found that not only direct contact of phosphopeptides with metal components, but also indirect contact with nanoLC pumps through the mobile phase causes significant losses during the recovery of phosphopeptides. Moreover, electrospray ionization was adversely affected by the mobile phase containing multiple metal ions as well as by the sample solvents contaminated with metal ions used in immobilized metal ion affinity chromatography for phosphopeptide enrichment. To solve these problems, metal ions were depleted by inserting an online metal ion removal device containing metal-chelating membranes between the gradient mixer and the autosampler. As a result, the peak areas of the identified phosphopeptides increased an average of 9.9-fold overall and 77-fold for multiply phosphorylated peptides with the insertion of the online metal ion removal system. This strategy would be applicable to the highly sensitive analysis of other phosphorylated biomolecules by microscale-LC/MS/MS.

## Highlights

• Development of bioinertized nano-LC/MS/MS for phosphopeptides.
• Metal ions in the mobile phases affect the phosphopeptide recovery.
• Metal ions in the mobile phases affect the ESI efficiency.
• Metal ion depletion was achieved by online metal-chelating membranes.
• Recovery of multiply phosphorylated peptides was 77-fold increased.

## Graphical Abstract

**Figure:**
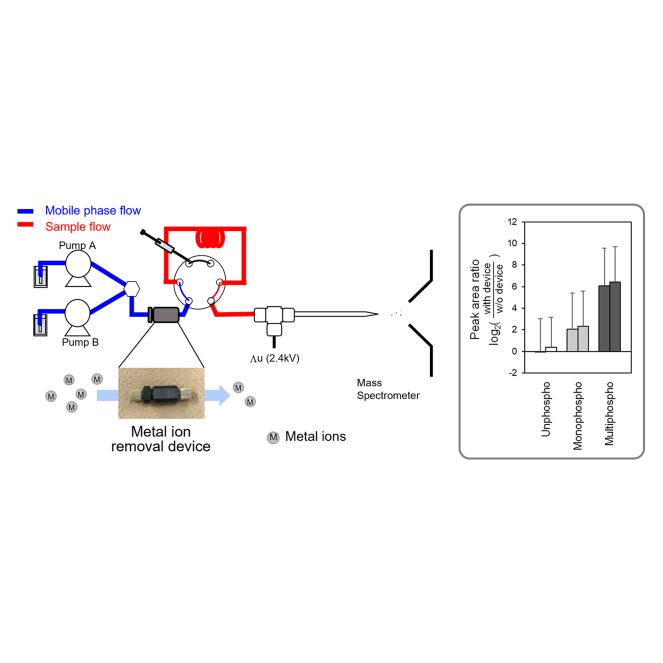

## In Brief

We developed a bioinertized nano-LC/MS/MS system using an online device containing metal-chelating membranes to increase the recovery of phosphorylated peptides. Using this method, the peak areas of the identified phosphopeptides increased an average of 9.9-fold overall and 77-fold for multiply phosphorylated peptides with the insertion of the online metal ion removal system.

Many kinds of phosphorylated biomolecules, including phosphoproteins, phospholipids, nucleic acids, and phosphorylated metabolites, are present in cells and are involved in key cellular functions, such as cell proliferation, protein synthesis, and metabolism (1, 2, 3, 4, 5). Therefore, quantitative analysis of phosphorylated biomolecules is essential to uncover their physiological functions and to elucidate the molecular basis of biological phenomena. In particular, protein phosphorylation causing abnormal activation is directly related to the pathogenesis of diseases such as cancer (6). To understand the mechanisms of these diseases, many researchers have searched for disease-specific phosphoproteins and phosphorylation sites (7, 8, 9, 10, 11, 12, 13). However, the amount of phosphopeptides present in digested peptides derived from human cell–extracted proteins is less than 0.5% (14) so that analysis requires highly sensitive analytical methods such as nanoflow LC/MS/MS (nanoLC/MS/MS) used in shotgun proteomics.

Phosphate groups are known to coordinate with various metal atoms and metal ions. Therefore, when metals such as stainless steel (SUS) are used in HPLC systems, distortion of peak shape and reduction in signal intensity occur (15, 16). To prevent interactions between metals and phosphate groups, the addition of EDTA, citric acid (17, 18, 19, 20), and phosphate (16, 21) has been employed. However, these additives suppress ionization in MS, and precipitation adversely affects both the column and the spray needle. To address these problems, columns made of metal-free bioinert materials, such as polyetheretherketone (PEEK), have been developed and are commercially available (22, 23), and new surface modification techniques have also been developed (24, 25).

Nevertheless, large-scale phosphopeptide analysis of trace samples using a combination of miniaturized LC, such as nanoLC and high-resolution MS, remains problematic. This is because nanoLC has a lower flow rate and narrower lines than conventional LC, resulting in a larger surface area per volume of the sample solution in contact with the inside surfaces of the lines. Furthermore, technological advances in nanoLC have led to the use of ultrahigh pressure, and as a result, new alloys and composite materials with high-pressure resistance are required for lines and valves. However, the interaction of these materials with phosphopeptides is unclear, and no studies have systematically investigated the effects of these materials on nanoLC/MS measurements.

In this study, approximately 3600 diverse phosphopeptides were extracted from human cultured cells and used as model substrates to investigate the effects of metal ions on sensitive quantification of protein phosphorylation in trace samples. Specifically, we examined the influence of components of the HPLC system that are in direct contact with the phosphopeptides and those that are not in direct contact with the phosphopeptides, such as pumps, on the analysis. In addition, the effect of metal ion contamination on ionization efficiency was examined. Based on the results, we constructed a bioinert nanoLC system by inserting an online metal ion removal device.

## Experimental Procedures

### Materials

Titanium dioxide (TiO_2_, titania) particles (Titansphere, particle size: 10 μm), Empore SDB-XC (Cat No 5065-64043), C18 (Cat No 5065-64041), C8 (Cat No 5065-64040), and Chelating Resin (Cat No 5065-64049) membrane disks were obtained from GL Sciences. Sequencing-grade modified trypsin was purchased from Promega. Water was purified with a Millipore Milli-Q system. Protease inhibitor cocktail, phosphatase inhibitor cocktail 2, and phosphatase inhibitor cocktail 3 were purchased from Sigma-Aldrich. All other chemicals were obtained from FUJIFILM Wako, unless otherwise specified.

### Preparation of Phosphopeptides From Cells

HeLa cells were cultured to 80% confluence in Dulbecco's modified Eagle’s medium containing 10% fetal bovine serum and 100 μg/ml kanamycin at 37 °C under 5% CO_2_ in 15 cm diameter dishes. The proteins were extracted as described previously (26). In short, the cell pellets were suspended in 100 mM Tris-HCl buffer (pH 9.0) containing 12 mM sodium deoxycholate (SDC), 12 mM sodium N-lauroylsarcosinate (SLS), protease inhibitor cocktail, and phosphatase inhibitor cocktail. The cells were incubated on a heating block at 95 °C for 5 min and then sonicated for 20 min. The extracted proteins were quantified using the BCA Protein Assay Kit, reduced with 10 mM dithiothreitol for 30 min, alkylated with 50 mM iodoacetamide for 30 min in the dark, diluted 5-fold with 50 mM ammonium bicarbonate, and digested with Lys-C for 3 h at room temperature and with trypsin for 16 h at room temperature. After digestion, 0.5% trifluoroacetic acid (TFA) was used to acidify the sample, and then an equal volume of ethyl acetate was added to remove the solubilizers SDC and SLS by extraction into the organic phase. Peptides in the aqueous phase were desalted using StageTips (27) packed with SDB-XC Empore Disks. Phosphopeptide enrichment was performed using the hydroxy acid-modified metal oxide chromatography (HAMMOC) method (28), which utilizes the chemical affinity between phosphate groups and TiO_2_. Briefly, a StageTip with a C8 Empore disk attached was equilibrated with a 0.1% TFA, 80% acetonitrile solution, and filled with TiO_2_ particles. Next, 300 mg/ml lactic acid, 0.1% TFA, and 80% acetonitrile solution were loaded into the TiO_2_-StageTip to coat the particle surface with lactic acid. Each sample was loaded onto a StageTip and the phosphopeptide was coordinated to the TiO_2_ particles. The phosphopeptides were then eluted with 0.5% piperidine solution after successive washes with 300 mg/ml lactic acid, 0.1% TFA, 80% acetonitrile solution, 0.1% TFA, and 80% acetonitrile solution, in that order. The eluate was acidified with 10% TFA to a final concentration of 0.1% and desalted using SDB-XC StageTips.

### Experimental Setup for Phosphopeptide Adsorption on HPLC Materials

To evaluate the adsorption of phosphopeptides on representative HPLC materials, we used inserts consisting of union connectors made from stainless steel, titanium, and PEEK (1/16" microvolume connectors, VICI). The HeLa phosphopeptides, enriched from 50 μg HeLa proteins, dissolved with 10 μl of the sample solvent (Solvent A, 4% acetonitrile and 0.5% TFA), was placed in an insert container, and allowed to stand for 5 min. Alternatively, the sample solvent was placed in the insert container for 5 min, and the collected solvent was used to prepare the HeLa phosphopeptide solution. These solutions were measured by nanoLC/MS/MS as described below.

### Collection of Mobile Phase Solutions for Preparation of Phosphopeptide Samples

In order to investigate the effect of the mobile phase on phosphopeptides, the mobile-phase solutions before (Presolv) and after (Postsolv) passage through the Ultimate 3000 RSLCnano pump (Thermo Fisher Scientific) were collected. For Presolv, the solution was collected from the bottle containing the mobile phase. For Postsolv, the solution was collected just after the gradient mixer of solutions A and B from pumps A and B. Metal ions in these mobile phases were quantified by ICPMS-2030 (Shimadzu).

### Preparation of Off-Line Micro-Tip Columns for Depleting Metal Ions

Pipette tip-based microcolumns for depleting metal ions were prepared using chromatographic sorbents extracted from a silica-based Ni-NTA Spin Column (QIAGEN). An Empore C8 membrane disk was hollowed out with an 18G flat-bottom needle and was used as a frit for a 200-μl pipette tip. The slurry solution of the Ni-NTA sorbents (20 mg/ml in methanol) was added to a 200-μl tip and packed by centrifugation. This method was based on the preparation of HAMMOC TiO_2_-StageTips described earlier.

### Fabrication of an Online Metal Ion Depletion Device

A custom-made online metal depletion device was fabricated by inserting a chelator membrane inside a PEEK inline filter (1/16-inch inner diameter, 0.5 μm mesh PEEK filter). The chelator membrane was made by hollowing out an Empore chelating disk with a 1/16-inch inner diameter flat-bottom needle. The online metal ion depletion device was installed between the nanoLC pump and the injector.

### NanoLC/MS/MS Measurement

NanoLC/MS/MS measurement of the HeLa phosphopeptides in Solvent A was performed with an Ultimate 3000 RSLCnano pump, an HTC-PAL (CTC Analytics,) autosampler with a Cheminert valve C72X (VICI), or an Inert ceramic angle valve (AMR) and a Q-Exactive mass spectrometer (Thermo Fisher Scientific). Phosphopeptides were separated on an in-house-pulled needle column (29) (150-mm length, 100 μm inner diameter, 6-μm needle opening) packed with ReproSil-Pur 120 C18-AQ 3-μm RP material (Dr Maisch). The LC mobile phases consisted of (A) 0.5% acetic acid and (B) 80% acetonitrile and 0.5% acetic acid. The injection volume was 5 μl, and the flow rate was 500 nl/min. Separation was achieved by applying a three-step linear gradient of 5 to 10% B in 5 min, 10 to 40% B in 20 or 60 min, 40 to 99% B in 5 min, and 99% B for 5 min. Spray voltage was set to 2.4 kV. Raw MS1 spectra were collected at a resolution of 70,000 with an automatic gain control target of 3 × 10^6^ and a maximum injection time of 100 ms. The MS1 mass range was set to 350 to 1500. The automatic gain control target value for MS2 was set at 1 × 10^5^, with a maximum injection time of 200 ms. The orbitrap was operated at 17,500 resolution, and higher energy collisional dissociation at a normalized collision energy of 27% was employed. The top five data-dependent MS2 scans were collected between full MS1 scans. All data were acquired in the profile mode using positive polarity.

### Data Analysis

Peak lists were created from the raw MS data using ProteoWizard (30) on the basis of the recorded fragmentation spectra. Peptides and proteins were identified by means of automated database searching using Mascot version 2.6.1 or 2.7.0 (Matrix Science) against the human proteome database from UniProtKB/Swiss-Prot (20,199 sequences released in 2017/04 or 20,380 sequences released in 2019/10) with a precursor mass tolerance of 5 ppm, a fragment ion mass tolerance of 20 ppm, and strict trypsin/P specificity allowing for up to two missed cleavages. Carbamidomethylation (C) was set as a fixed modification. Methionine oxidation and phosphorylation on serine, threonine, and tyrosine were allowed as variable modifications. Peptides were accepted if the Mascot score was over the 95% confidence limit (*p* < 0.05) based on the identity score of each peptide. False discovery rates of less than 1%, estimated by searching against a reversed decoy database, were confirmed at a peptide-spectrum match level. Label-free quantitation was performed by Thermo MSFileReader based on integrating signal intensities over time for each peak on the extraction ion chromatogram. Match-between runs for each experiment were performed by an in-house script in which the retention times of the two runs were aligned by linear interpolation of the commonly identified peptides.

### Direct Infusion-MS Measurement

Samples were continuously delivered into the mass spectrometer using the direct infusion method by a syringe pump equipped with a TripleTOF 5600+ mass spectrometer (SCIEX). Synthetic peptides were obtained from Synpeptide (Shanghai, PRC). The peptide sample dissolved in 10% acetonitrile and 0.5% acetic acid was aspirated using a 500 μl Hamilton syringe and delivered through a 75 μm i.d. fused silica capillary at 1 μl/min with the syringe pump. A home-made electrospray needle (tip opening diameter: 10 μm) with a spray voltage of +2.4 kV was used for electrospray ionization (ESI)-MS analysis. Four different metal ions (FeCl_2_, NiCl_2_, ZnCl_2_, and Ti(SO_4_)_2_) were spiked to the sample solution at 5 μM. The flow path was washed with 5 mM citric acid solution between measurements.

### Experimental Design and Statistical Rationale

For each experiment, a pooled phosphopeptide mixture was used for samples to exclude any sources of variation in sample preparation. Therefore, since the only difference between samples was the sample solvent composition, each sample was prepared with n = 1, and duplicate LC/MS/MS measurements were performed for each sample. Control samples were prepared by dissolving the pooled phosphopeptides in Solvent A. Direct injection-MS measurements were performed four times per condition, and the average intensity of each measurement was calculated. In the case of the online metal ion removal device experiment, the condition without the device was used as control, and the identical sample solution was used.

## Results and Discussion

### Evaluation of Adsorption of Phosphopeptides on HPLC Materials

**Fig. 1:**
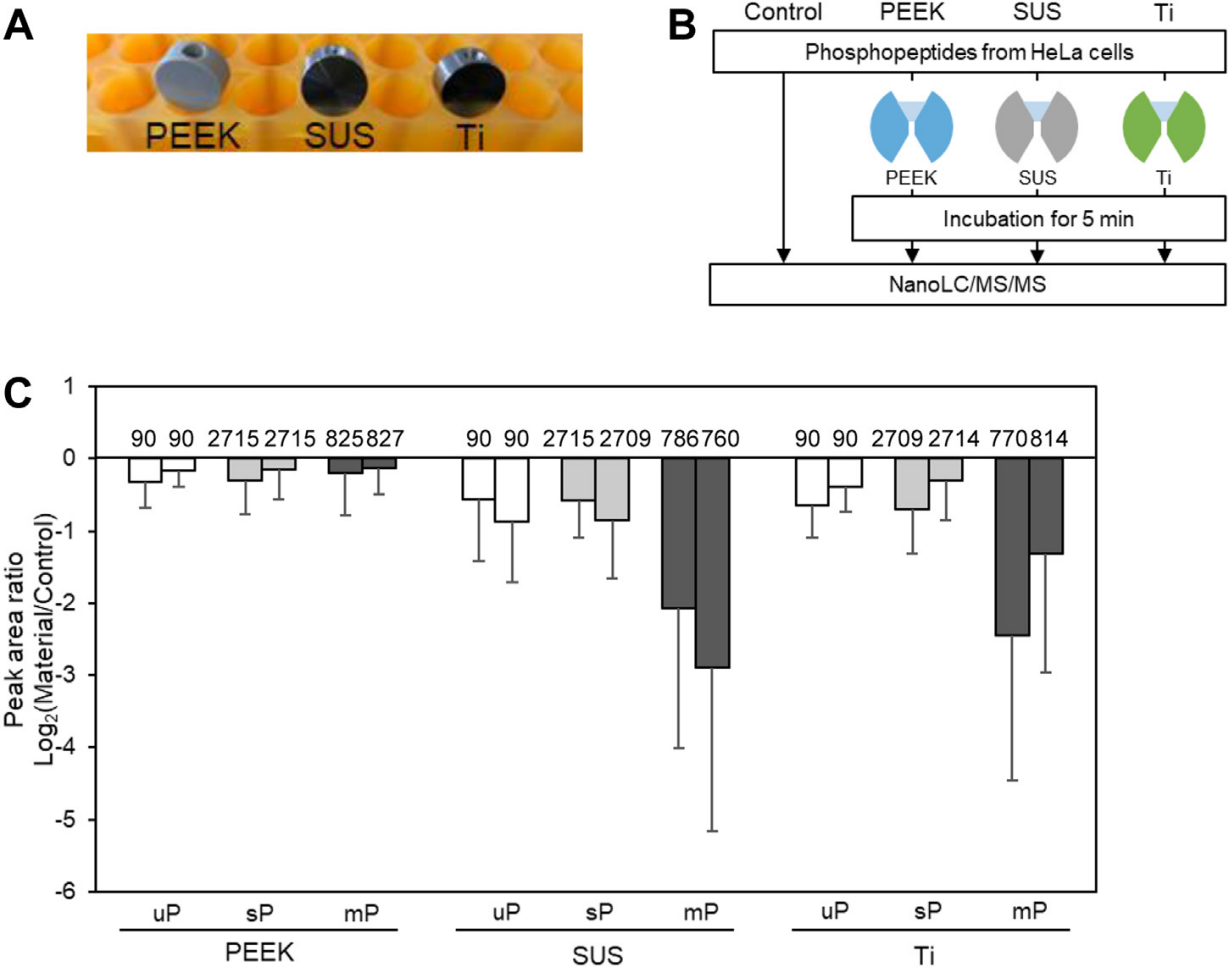
**Evaluation of adsorption on union materials.***A*, union connectors used for evaluation (PEEK, SUS, and Ti). *B*, workflow for evaluating phosphopeptide adsorption on union materials. *C*, the bar plots show the peak area Log_2_ ratio of Material to Control for commonly identified peptides. Duplicate nanoLC/MS/MS measurements were performed for each sample as shown by paired bars. The error bar shows the standard deviation of the peak area ratios for different peptides. The number on each bar indicates the number of identified peptides after MBR treatment. mP, multiply phosphorylated peptides; sP, singly phosphorylated peptides; uP, unphosphorylated peptides.

To evaluate the adsorption of phosphopeptides in HPLC systems, we selected three materials commonly used as transfer tube connection unions: SUS, titanium (Ti), and PEEK (Fig. 1, *A* and *B*). The test sample was prepared from 50 μg of tryptic digest of proteins extracted from HeLa cells, and approximately 250 ng of phosphopeptides was obtained by titanium dioxide chromatography. In total, 3632 phosphopeptides were identified and quantified by nanoLC/MS/MS measurement after applying the match-between-runs treatment. These peptides have a wide range of physical properties, with molecular weights ranging from 800 to 5000, residues from 7 to 46, and hydrophobicity index (gravy score) from −3.4 to 1.0. The number of phosphate groups per peptide ranges from 1 to 4, with an average of 1.3. The isoelectric points are distributed from 2.5 to 12.5. This mixture of phosphopeptides with a wide range of properties was evaluated for adsorption on the above three materials. Specifically, as shown in Figure 1*B*, the phosphopeptide solution was placed in the conical hollows of the three unions for 5 min, and then the solution was collected for nanoLC/MS/MS measurement. As shown in Figure 1*C*, the overall recovery was lower for both SUS and Ti unions, and this trend was especially significant for the more strongly retained hydrophobic peptides (supplemental Fig. S1). Furthermore, multiply phosphorylated peptides with two or more phosphate groups showed 4.3- and 5.4-fold higher decreases for both SUS and Ti unions, respectively. On the other hand, the recovery was close to 100% for the PEEK union. These results show that phosphopeptides are easily adsorbed on metal materials such as SUS and Ti to an extent that depends on their physical properties. The analytical system was found to be suitable for quantitative evaluation of the adsorption behavior of phosphopeptides under various conditions.

### Recovery of Phosphopeptides in Solvents Taken From Metal Union Connectors

**Fig. 2:**
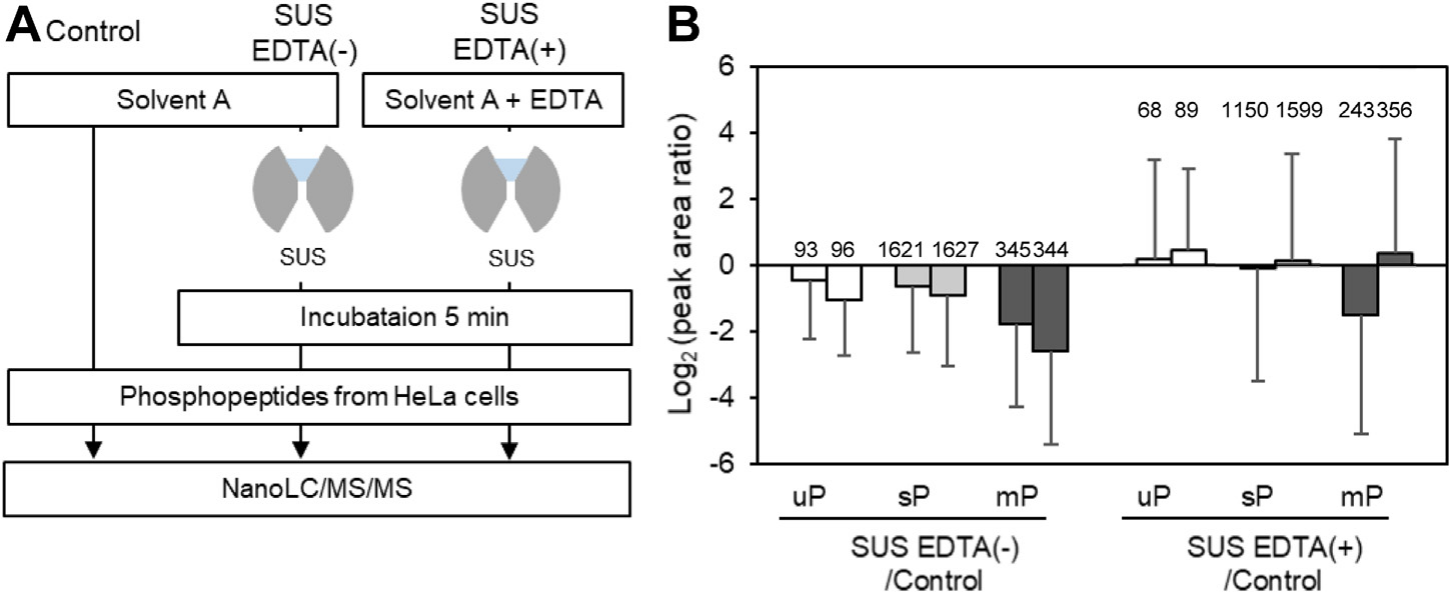
**Evaluation of interaction between phosphopeptides and metal ions eluted from metal connectors.***A*, workflow for evaluation of adsorption of phosphopeptides using solvent A in contact with SUS and the effect of EDTA. *B*, the bar plots show the peak area Log_2_ ratio of SUS EDTA(−) to Control or SUS EDTA(+) to Control for commonly identified peptides. The error bar shows the standard deviation of the peak area ratios for different peptides. Duplicate nanoLC/MS/MS measurements were performed for the SUS EDTA(−) condition, while 500 nM (*left bar*) and 50 μM (*right bar*) EDTA solutions were analyzed for the SUS EDTA(+) condition as shown by paired bars. The number on each bar indicates the number of identified peptides after MBR treatment. mP, multiply phosphorylated peptides; sP, singly phosphorylated peptides; uP, unphosphorylated peptides.

In this experiment, sample solutions were prepared using solvents taken from the metal union connectors, and the effect on the recovery when the sample is not in direct contact with the metal was examined. The workflow is shown in Figure 2*A*. At first, Solvent A only was placed in the SUS union connector and allowed to stand for 5 min before collection. The phosphopeptides were dissolved in the solution and were analyzed by nanoLC/MS/MS (SUS EDTA(−)). The same procedure was also performed for Solvent A but in the presence of 500 nM or 50 μM EDTA (SUS EDTA(+)). As a result, a 50% decrease on average in the recovery of identified peptides was observed even when the phosphopeptides were not in direct contact with SUS (Fig. 2*B*). This trend was especially significant for multiply phosphorylated peptides. The addition of EDTA to Solvent A was effective to improve the recovery, indicating that metal ions eluted from the metal connectors interact with phosphopeptides, resulting in lower recovery. EDTA in the sample solvents also prevents the interaction between metal ions in the nanoLC/MS/MS system and phosphopeptides. Furthermore, the repeatability of nanoLC/MS/MS measurements was examined for Control and SUS EDTA(−), respectively (supplemental Fig. S2). As a result, the reproducibility of SUS EDTA(−) was lower than that of Control, indicating that metal ions in the sample solvent affect not only the recovery of peptides but also the reproducibility.

### Investigation of Adsorption Effect Using Mobile Phase With Metal Ions Eluted From the nanoLC System

**Fig. 3:**
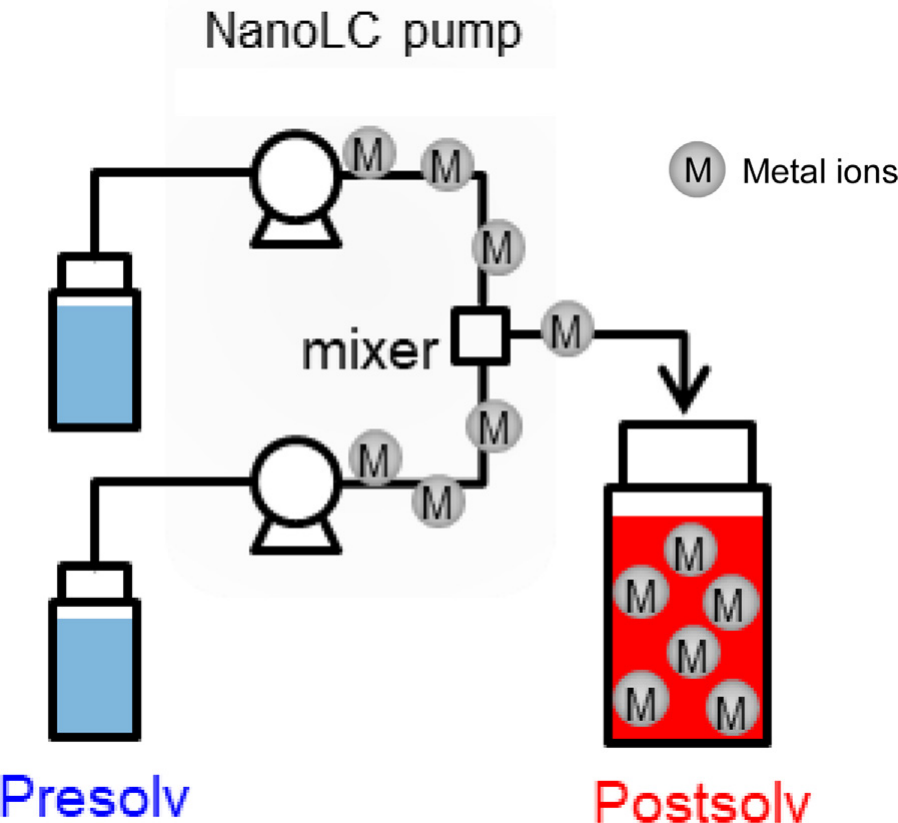
**Detection of trace metals in the mobile phase.** Schematic representation of collecting sites of the mobile phase. Presolv is the mobile phase that has not passed through the nano-LC pump. Postsolv is the mobile phase that has passed through the nano-LC pump.

In order to investigate the influence of the solution flowing through the nanoLC system as well as the aforementioned SUS union connector, the mobile phase taken from the actual nanoLC system was used as the sample solvent. Figure 3 shows the location of the mobile phase collection. Prior to the adsorption experiment, metal ions in the mobile phase that had passed through the pump and mixer (Postsolv) were quantified by ICP/MS. As a result, it was found that not only iron, nickel, and chromium ions, which are known to be contained in SUS, but also various other metal ions such as zinc, titanium, and vanadium were present above 1 ppb. The detected metal ions and their contents were almost the same as previously reported (31, 32).

**Fig. 4:**
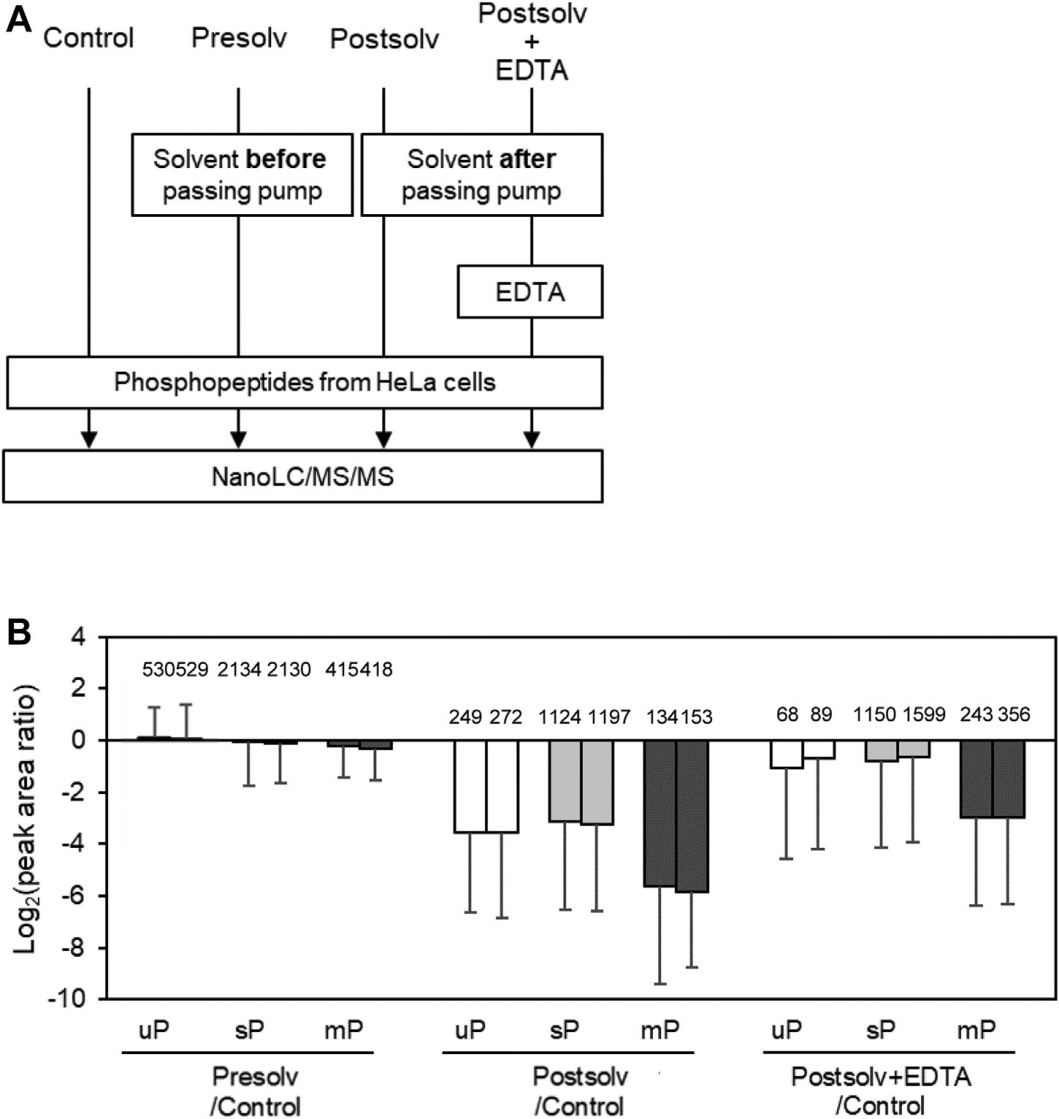
**Adsorption effects using mobile phase-derived solvents.***A*, workflow for evaluating the effect of metal ions eluted before and after passage through the nanoLC pump and recovery with the addition of EDTA. *B*, the bar plots show the peak area Log_2_ ratio of Presolv to Control, Postsolv to Control or Postsolv +500 nM EDTA to Control for commonly identified peptides. Duplicate nano-LC/MS/MS measurements were performed for each sample as shown by paired bars. The error bar shows the standard deviation of the peak area ratios for different peptides. The number on each bar indicates the number of identified peptides after MBR treatment. mP, multiply phosphorylated peptides; sP, singly phosphorylated peptides; uP, unphosphorylated peptides.

We then concentrated Postsolv 100-fold and evaluated its effect on the recovery of phosphopeptides when it was used as the sample solvent (Fig. 4*A*). For comparison, a sample using a mobile phase (Presolv) that had not passed through the nanoLC pump as a solvent was subjected to nanoLC/MS/MS. Figure 4*B* shows that the peak areas of the commonly identified peptides from these two samples are almost identical. In contrast, the peak area of phosphopeptides was reduced by an average 90% for the sample with Postsolv as the solvent. In particular, the recovery of multiply phosphorylated peptides was significantly lower, compared to the Control (1.9%). However, the addition of 500 nM EDTA to Postsolv improved the recovery of phosphopeptides, but had little effect on some phosphopeptides, especially multiply phosphorylated peptides, owing to the low concentration of EDTA. These results suggest that metal ions in the mobile phase also affect the recovery of phosphopeptides.

### Effect of Chelating Resins on the Removal of Metal Ions in the Mobile Phase

As mentioned earlier, EDTA is effective in improving the recovery of phosphopeptides, but its addition to the mobile phase is known to cause the clogging of analytical columns and sprays due to precipitation (17). It also suppresses ionization in ESI-MS (19). As an alternative to EDTA, chelating resins were considered here to remove metal ions in the mobile phase.

**Fig. 5:**
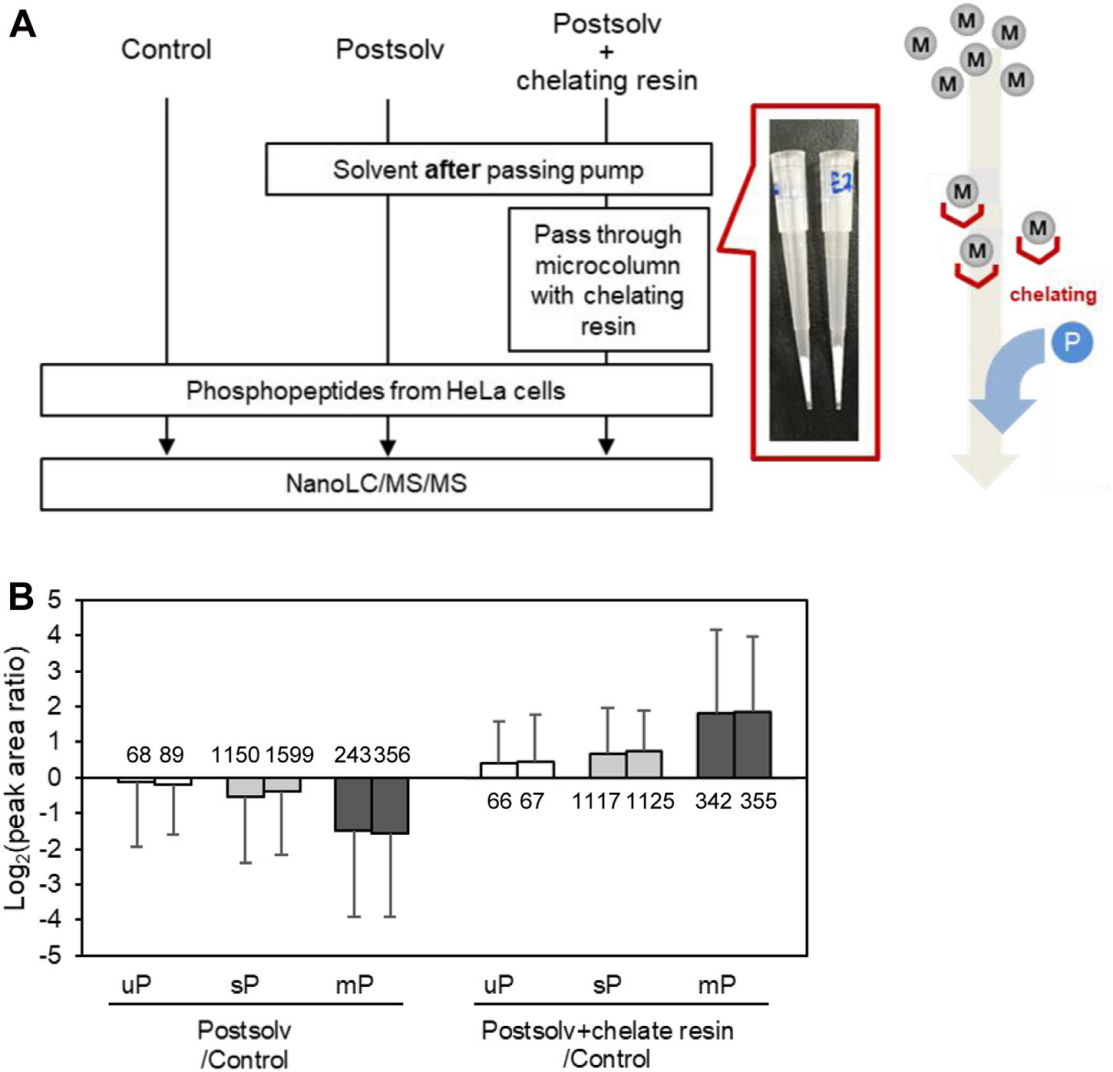
**Removal of metal ions from the mobile phase using chelating resin-filled chips.***A*, schematic representation of workflow. Off-line metal ion removal by chelating resin. *B*, the bar plots show the peak area Log_2_ ratio of Postsolv to Control or Postsolv + chelating resin to Control for commonly identified peptides. Duplicate nano-LC/MS/MS measurements were performed for each sample as shown by paired bars. The error bar shows the standard deviation of the peak area ratios for different peptides. The number on each bar indicates the number of identified peptides after MBR treatment. mP, multiply phosphorylated peptides; sP, singly phosphorylated peptides; uP, unphosphorylated peptides.

The workflow is shown in Figure 5*A*. As in the previous experiment, the peak area of phosphopeptides showed a decreasing trend when Postsolv was used as a solvent compared to the solvent-untreated sample (Control) (Fig. 5*B*). On the other hand, Postsolv was purified by a mini-column packed with chelating resin and used as the sample solvent (Postsolv + chelating resin), the peak areas of phosphopeptides were improved to even higher levels than the Control by passing the mobile phase-derived solvent through the chelating resin (Fig. 5*C*). This might be because the trace amounts of metal ions contained in the Control solvent were also depleted by the chelating resin. Note that depletion of metal ions by NTA-based resin is advantageous for MS analysis because no chelating agent remains in solution, which is not the case with EDTA.

### Insertion of an Online Metal Ion Removal Device Into the nanoLC System

**Fig. 6:**
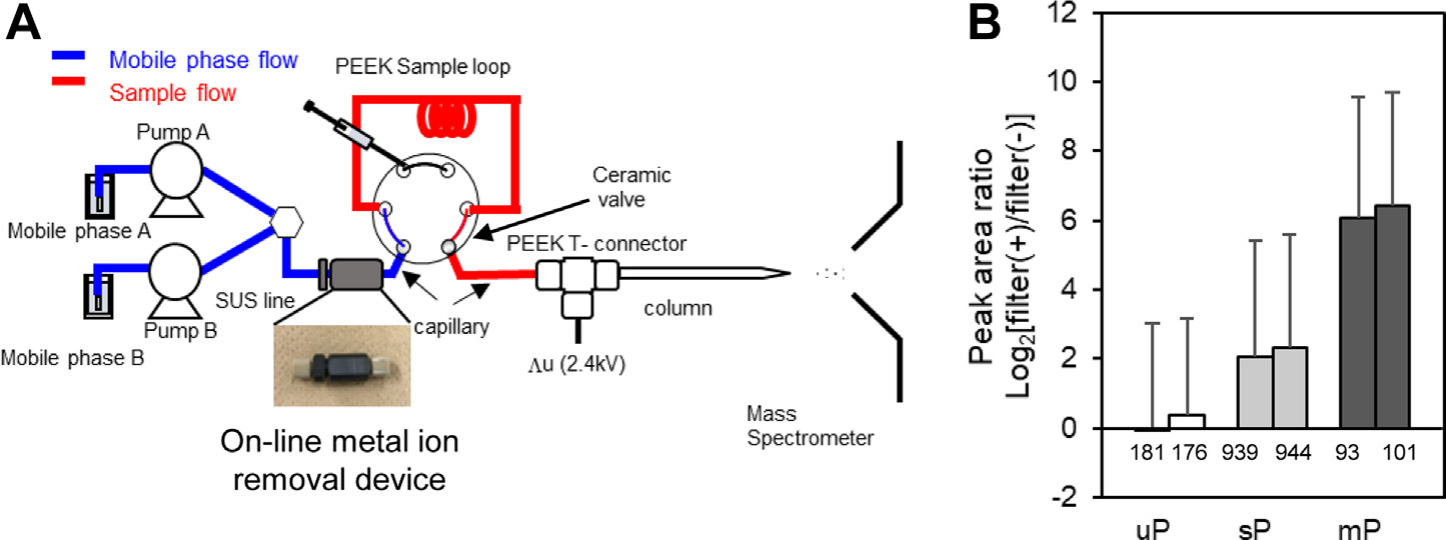
**Removal of metal ions from nanoLC systems using an online metal ion removal device.***A*, schematic representation of workflow. To make the flow line after the online device metal-free, a fully inert system was developed using ceramic valves, PEEK sample loops, capillary lines, PEEK T-union connectors for voltage application, and the nanocolumn with self-assembled particle frits. *B*, the bar plots show the peak area Log_2_ ratio of with the online device to without the online device for commonly identified peptides. The error bar shows the standard deviation of the peak area ratios for different peptides. The number on each bar indicates the number of identified peptides after MBR treatment. mP, multiply phosphorylated peptides; sP, singly phosphorylated peptides; uP, unphosphorylated peptides.

Since offline chelating resin columns were effective for metal ion depletion, an online metal depletion system using chelating resin columns was next prepared by inserting an Empore iminodiacetate (IDA)-immobilized membrane disk into a PEEK union (6.25 μl capacity) and placed between the nanoLC pump and injector. The system is shown in Figure 6*A*. To clarify the effect of the on-line metal ion removal device, a SUS line was used upstream of the online device, and metal-free PEEK and fused silica capillaries were used downstream of the on-line device.

When we compared the effect of the online device using a sample of phosphopeptides derived from HeLa cells, the peak areas of identified phosphopeptides were increased by an average of 9.9-fold by inserting the online metal ion removal device (Fig. 6*B*). For multiple phosphorylated peptides in particular, peak areas increased an average of 77-fold. Although the reproducibility did not change with or without the on-line device, the peak area was greatly improved regardless of the amount of phosphopeptides (supplemental Fig. S4). These results indicate that the on-line device is useful for improving the recovery of phosphopeptides.

### Effect of Metal Ions in ESI

Since the nanoLC mobile phase contains many metal ions and the sample solvent may contain metal ions derived from immobilized metal ion affinity chromatography (IMAC), which is commonly used in phosphopeptide sample preparation, we next investigated the effect of these metal ions on ESI efficiency. To investigate the effect on ionization efficiency alone, ESI-MS measurements were performed in the direct infusion mode using a synthetic peptide as the sample. Four synthetic phosphopeptides were selected as highly adsorbable sequences based on previous studies using phosphopeptide samples derived from HeLa cells, and two unphosphorylated peptides were selected as controls.

Metal ions evaluated were FeCl_2_, FeCl_3_, NiCl_2_, ZnCl_2_, and Ti(SO_4_)_2_, which were detected by ICP/MS at particularly high concentrations. The concentration of metal ions was set at 50 nM to match the concentration detected by ICP/MS. However, under these conditions, the measurements were not stable and the decrease in ionization efficiency could not be observed reproducibly.

On the other hand, immobilized metal ion affinity chromatography, IMAC (28, 33, 34, 35), using a resin with various immobilized metal ions, such as titanium, zirconium, and iron, as the stationary phase is used to enrich phosphopeptides. Phosphopeptides concentrated by this method may be contaminated with μM levels of metal ions. Therefore, we investigated the effect of the addition of 5 μM metal salts to the above synthetic peptides on ionization efficiency.

**Fig. 7:**
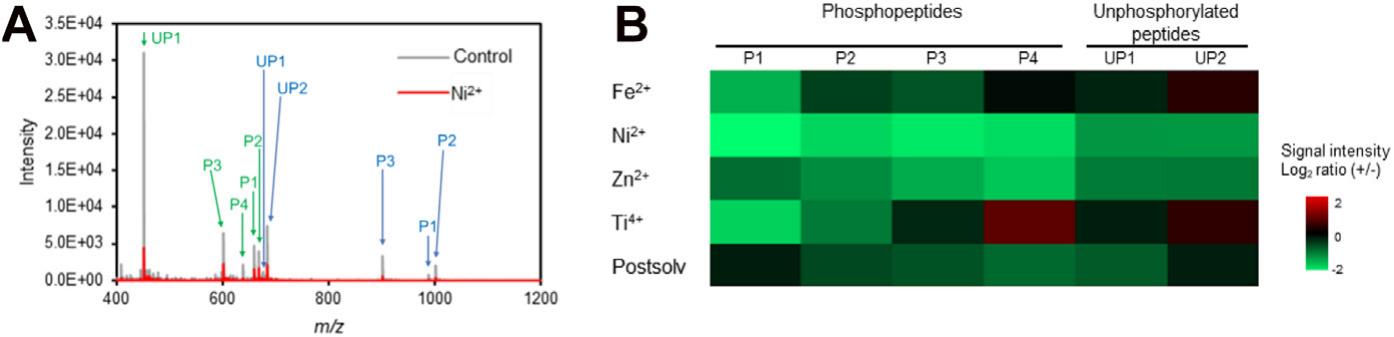
**Effect of metal ions on ESI sensitivity of peptides.***A*, mass spectrum of a mixture of six synthetic peptides in the presence or absence of 5 μM Ni^2+^ ions. Annotation in *green* represents triply charged peptide ions whereas annotation in *blue* represents doubly charged ions. Peptides: (P1) KAEDpSDpSEPEPEDNVR, (P2) IGDEYAEDpSpSDEEDIR, (P3) SSGpSEpTEQVVDFSDR, (P4) NDpSWGSFDLR, (UP1) Ac-QALDDSVKAHAR-amide, (UP2) Ac-EDDAGSEFGEDR-amide. *B*, signal intensity ratio of the six synthetic peptides in the presence of various metal ions with respect to the Control. Quadruplicate measurements for each condition were conducted. ESI, electrospray ionization.

Figure 7*A* shows the mass spectrum upon the addition of Ni^2+^ ions. The signal intensity of the phosphopeptide was decreased by Ni^2+^ ions. Similarly, the signal intensity decreased upon the addition of other metal ions (Fig. 7*B*). The signal intensity also showed a decreasing trend when Fe^3+^ was added, but the extent of the decrease was unclear due to the instability of the experimental system (data not shown). The effect on ionization efficiency varied depending on the metal ion species and peptide sequence. Furthermore, the signal intensity decreased when the mobile phase-derived Postsolv was collected and used at its original concentration.

These results suggest that the presence of high concentrations of metal ions in a sample not only reduces sensitivity due to adsorption but also affects the ionization efficiency in MS. Although no reproducible decrease in ionization efficiency was observed with the addition of about 50 nM of each metal salt, it was confirmed that even a metal salt concentration of 1∼10 ppb can affect the ionization efficiency when multiple metal species are present, as in the case of mobile phase-derived Postsolv.

## Conclusion

In this study, we developed a bioinertized nanoLC/MS/MS system for the accurate and sensitive quantification of phosphopeptides. We found that phosphopeptides are not only adsorbed on metal components through direct contact, but also they interact with metal ions derived from components with which they are not in direct contact such as pumps. In addition, we have shown that MS ionization efficiency is reduced when using mobile phases containing multiple metal ions or when using samples containing several μM metal ions released during phosphopeptide enrichment processes such as IMAC. To prevent such interactions between metal ions and phosphopeptides, we aimed to deplete metal ions from the mobile phases. Indeed, the use of an online metal ion removal device packed with a membrane containing chelating resins improved both the identification number and the recovery of phosphopeptides. This analytical strategy is expected to be useful not only for phosphopeptides but also for other phosphorylated biomolecules and should be widely applicable for highly sensitive analysis using miniaturized LC coupled with ESI/MS.

## Data Availability

All MS raw files as well as the peak picking and the result files were deposited at a public repository of ProteomeXchange Consortium (http://proteomecentral.proteomexchange.org) *via* the jPOST partner repository (https://jpostdb.org) (36) with the data set identifier PXD034942 in Complete Submission format.

For reviewers only

https://repository.jpostdb.org/preview/17685959746384aeee2b61d Access key: 2912

## Supplemental data

This article contains supplemental data.

## Conflict of interest

The authors declare no competing interests.
