# Supplementary material for: Bioinertization of NanoLC/MS/MS Systems by Depleting Metal Ions From the Mobile Phases for Phosphoproteomics
Source: Mol Cell Proteomics. 2023 Mar 22;22(5):100535. doi: 10.1016/j.mcpro.2023.100535 (PMC10172917; doi:10.1016/j.mcpro.2023.100535)
Supplement: Supplemental Figures S1–S4 and Table S1 Legend [file mmc1.docx]

**Supplemental Data**

**Bioinertization of nanoLC/MS/MS systems by depleting metal ions from the mobile phases for phosphoproteomics**

Yumi Komori^1^, Tomoya Niinae^1^, Koshi Imami^1, 2^, Jun Yanagibayashi^3^, Kenichi Yasunaga^3^, Shinya Imamura^3^, Masami Tomita^3^ and Yasushi Ishihama^1,4＊^

1) Graduate School of Pharmaceutical Sciences, Kyoto University, Kyoto 606-8501, Japan.

2) ​RIKEN Center for Integrative Medical Sciences, Tsurumi-ku, Yokohama, Kanagawa 230-0045, Japan.

3) Shimadzu Corporation, Kyoto 604-8511, Japan.

4) Laboratory of Clinical and Analytical Chemistry, National Institute of Biomedical Innovation, Health and Nutrition, Ibaraki, Osaka, 567-0085, Japan.

*Corresponding author:

Tel: +81-75-753-4555, Fax: +81-75-753-4601, E-mail: yishiham@pharm.kyoto-u.ac.jp

--------------------------------------------------------------------------------------------------------------------

**Table of contents**

**Figure S1.** Evaluation of adsorption on union materials.

**Figure S2.** Effect of metal ions eluted from metal connectors on the reproducibility of nanoLC/MS/MS measurements.

**Figure S3.** Removal of metal ions from the mobile phase using chelating resin-filled tips.

**Figure S4.** Repeatability in nanoLC/MS/MS systems with or without on-line metal ion removal device**.**

**Table S1.** Identified and quantified phosphopeptides for all figures.


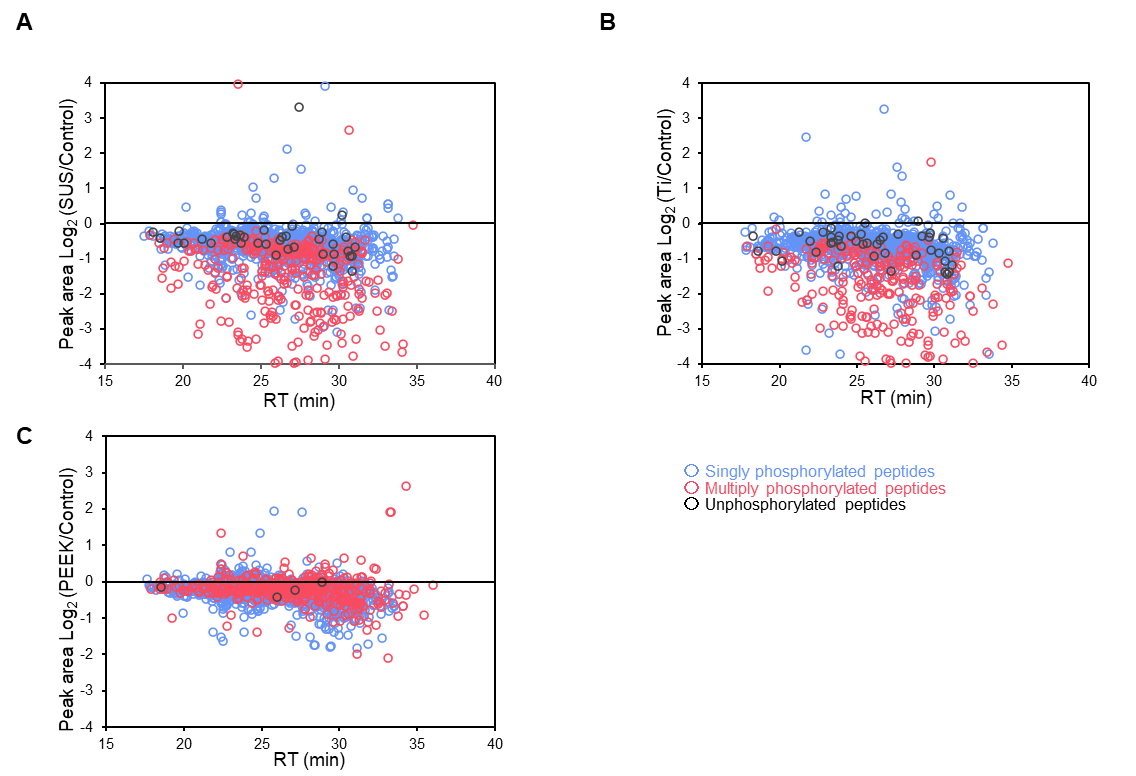


**Figure S1. Evaluation of adsorption on union materials.** (A) Dependence of peak area Log_2_ ratio of SUS to Control for commonly identified peptides (n = 1,793) on the retention times (RT) of nanoLC/MS/MS. (B) Dependence of peak area Log_2_ ratio of Ti to Control for commonly identified peptides (n = 1,790) on RT of nanoLC/MS/MS. (C) Dependence of peak area Log_2_ ratio of PEEK to Control for commonly identified peptides (n = 1,859) on RT of nanoLC/MS/MS.


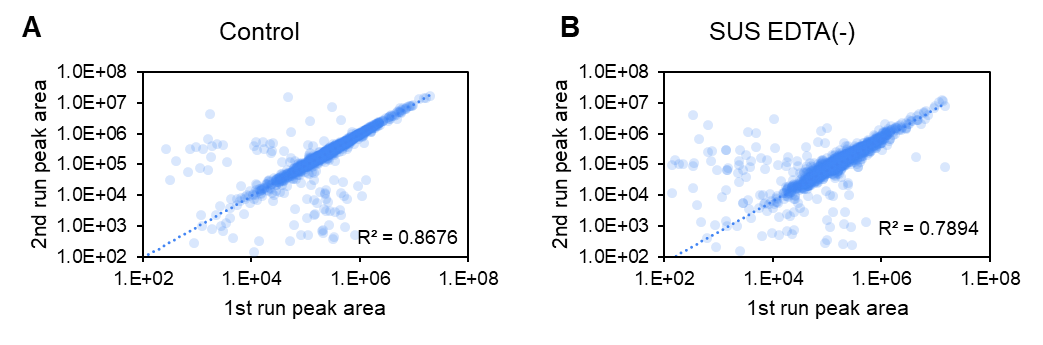


**Figure S2. Effect of metal ions eluted from metal connectors on the reproducibility of nanoLC/MS/MS measurements.** (A) Correlation plot between the first (x-axis) and second (y-axis) measurements for Control, (B) correlation plot between the first (x-axis) and second (y-axis) measurements for SUS EDTA(-).


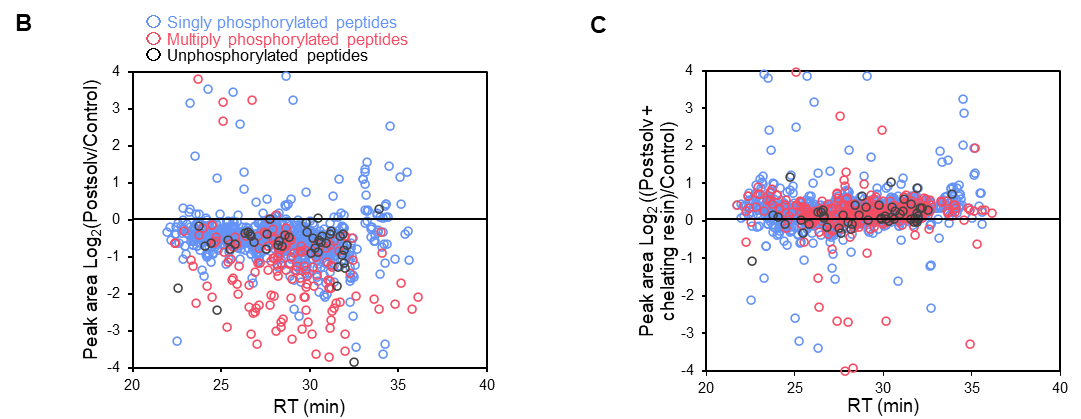


**B**

**A**

**Figure S3. Removal of metal ions from the mobile phase using chelating resin-filled tips.** (A) Dependence of peak area Log_2_ ratio of Postsolv to Control for commonly identified peptides (n = 1,088) on RT of nanoLC/MS/MS. (B) Dependence of peak area Log_2_ ratio of Postsolv + chelating resin to Control for commonly identified peptides (n = 1,450) on RT of nanoLC/MS/MS.


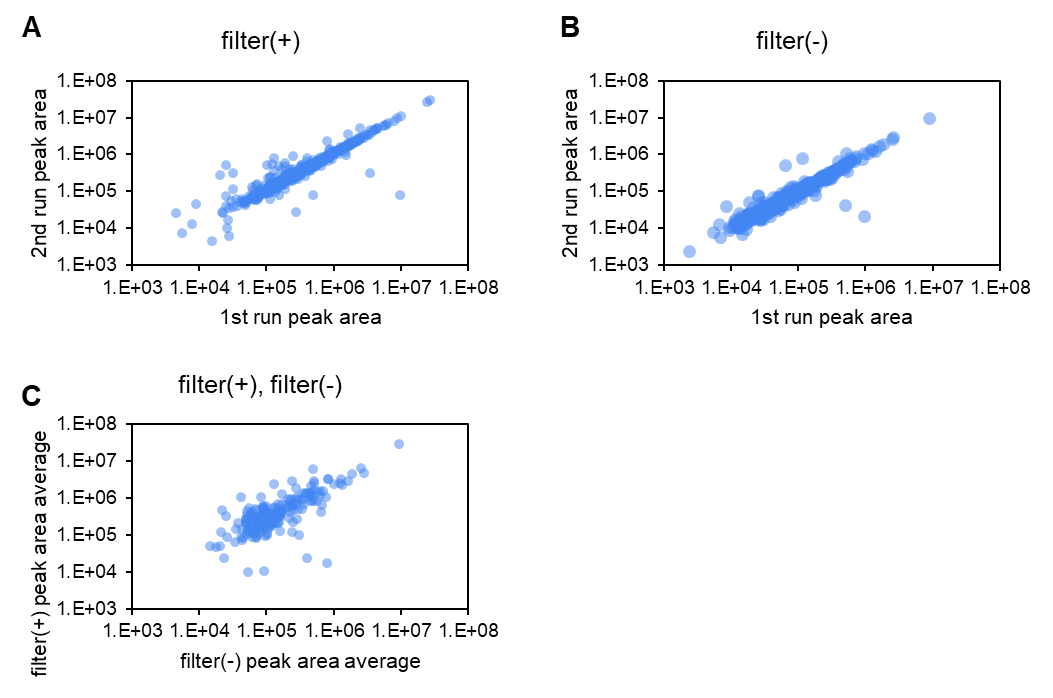


**Figure S4. Repeatability in nanoLC/MS/MS systems with or without on-line metal ion removal device.** (A) Correlation plot between the first (x-axis) and second (y-axis) runs with the on-line device. (B) Correlation plot between the first (x-axis) and second (y-axis) runs without the on-line device. (C) Correlation plot between average peak area without the on-line device (x-axis) and average peak area with the on-line device (y-axis).
